# Photoreactivity of an Exemplary Anthracene Mixture Revealed by NMR Studies, including a Kinetic Approach

**DOI:** 10.3390/molecules26216695

**Published:** 2021-11-05

**Authors:** Kristina Kristinaityte, Mateusz Urbańczyk, Adam Mames, Mariusz Pietrzak, Tomasz Ratajczyk

**Affiliations:** Institute of Physical Chemistry, Polish Academy of Sciences, PL-01224 Warsaw, Poland; kkristinaityte@ichf.edu.pl (K.K.); murbanczyk@ichf.edu.pl (M.U.); amames@ichf.edu.pl (A.M.)

**Keywords:** anthracene, UV-illumination, photodimerization, NMR, kinetics

## Abstract

Anthracenes are an important class of acenes. They are being utilized more and more often in chemistry and materials sciences, due to their unique rigid molecular structure and photoreactivity. In particular, photodimerization can be harnessed for the fabrication of novel photoresponsive materials. Photodimerization between the same anthracenes have been investigated and utilized in various fields, while reactions between varying anthracenes have barely been investigated. Here, Nuclear Magnetic Resonance (NMR) spectroscopy is employed for the investigation of the photodimerization of two exemplary anthracenes: anthracene (**A**) and 9-bromoanthracene (**B**), in the solutions with only **A** or **B**, and in the mixture of **A** and **B**. Estimated *k* values, derived from the presented kinetic model, showed that the dimerization of **A** was 10 times faster in comparison with **B** when compounds were investigated in separate samples, and 2 times faster when compounds were prepared in the mixture. Notably, the photoreaction in the mixture, apart from **AA** and **BB**, additionally yielded a large amount of the **AB** mixdimer. Another important advantage of investigating a mixture with different anthracenes is the ability to estimate the relative reactivity for all the reactions under the same experimental conditions. This results in a better understanding of the photodimerization processes. Thus, the rational photofabrication of mix-anthracene-based materials can be facilitated, which is of crucial importance in the field of polymer and material sciences.

## 1. Introduction

Polycyclic aromatic hydrocarbons, commonly known as acenes, are a significant class of organic molecules built from fused benzene rings which are arranged linearly [1]. Their interesting chemical and physical properties have attracted the attention of researchers, and many various organic synthetic routes, as well as experimental and theoretical studies, have been devoted to this class of organic compounds [2]. Moreover, acenes are widely utilized in many branches of material sciences and medicine [3,4,5].

Among acenes, anthracene and its derivatives are of central interest, which is primarily due to their relatively easy synthesis and purification, which facilitates their potential applications in various fields of chemistry, material sciences and biology [6,7,8]. In particular, one of the most important research issues regarding anthracenes is related to their photoreactivity, which, in principle, involves two scenarios of [4 + 2] and [4 + 4] photoinduced cycloadditions [9,10,11].

Regarding the [4 + 2] photoinduced scenario, the reaction of anthracenes with singlet oxygen is the most investigated issue, as this reaction yields endoperoxides (EPOs), which are of central importance in many fields [11,12,13,14]. For example, EPOs are used in organic synthesis for the generation of hydrogen peroxide under mild conditions [15]. In medicine and biology, the cycloaddition of oxygen to anthracenes can be used in photodynamic therapy in order to combat malignant cells with a controlled release of oxygen species [16], for detecting singlet oxygen in biological systems [17], and for optical imaging [18].

Concerning the [4 + 4] scenario, the photochemistry of anthracene dimerization is of central interest. In principle, the photodimerization occurs upon irradiation in two different wavelength (*λ*) windows. The less energetic *λ* (>300 nm) is usually chosen for photodimerization in order to avoid a competitive photodissociation process, which occurs at a shorter *λ* (<300 nm) regime [18,19]. Recently, there has been a growing interest in shifting the absorption band to an even higher *λ* regime [18,19] in order to avoid damage to the surrounding matrix and biological tissues caused by UV light. In the precise wavelength-dependent study, Kislyak et al. [19] presented a detailed photokinetic framework highlighting the importance of the initial conditions for the reaction pathway.

Regarding the reaction pathway, it is unquestionable that the singlet excited state is involved in the reaction mechanism. For example, one of the possible mechanisms of anthracene dimerization involves three steps. At first, the anthracene molecule is excited, then a pair of excited and unexcited molecules is formed. In the third step, this pair reacts, forming a dimer [19,20]. However, fluorescence is possible, and the excimer can split up into two unexcited molecules. In this context, dimerization and fluorescence are two competitive processes. This basic scenario can sometimes be more complex and can involve various transient species. For example, many issues concerning the excimer are still under investigation [21]. The issue of the photodimerization of anthracenes has been addressed many times. Many studies have been devoted to the kinetics and reactivity of substituted anthracenes, which depends on the electronic properties of the substituent. Furthermore, different isomers of the dimer can be formed [9,22,23]. Therefore, the regio- and enantioselectivity of anthracene photodimerization has been investigated comprehensively, both via experiment and quantum chemistry calculations [24,25,26,27,28].

Regarding the applications of anthracenes, tunable luminescence and easy charge transfer coupled with the unique ability of the reversible dimerization make anthracenes valuable building blocks for the generation of light responsive materials [9,29,30]. This includes various polymeric materials [30,31], such as intelligent shape memory polymers [32], as well as soft materials, such as photoresponsive hydrogels [16] and liquid crystals [33]. Hence, the main applications cover a vast number of fields, including tissue engineering [30], optoelectronic devices [33] such as organic light-emitting diodes or transistors [34], sensors for metal ion detection [35], molecular containers for catalysis or drug delivery [36] and photomedicine, where these compounds can be used as intracellular pH probes [37], DNA-based hybridization sensors [38] and other biosensors [39]. According to the latest results, anthracene derivatives have even been suggested as potential candidates for applications in laser photonics [40] and as smart optical materials [41].

Taking into account the vast majority of data concerned with the photochemistry of anthracenes, one can notice that previous studies and applications have focused on the photoreactions leading to homodimers, i.e., the dimers created from two identical monomer molecules. Interestingly, a few examples of photoreactions between anthracenes and higher acenes have already been presented, but no comprehensive kinetics have been mentioned [6]. This is quite an interesting situation, as the reaction between different anthracenes raises many interesting questions. It is intriguing also from the application point of view, as a more complex and functional systems may have the potential to be photo fabricated.

Mixtures containing various anthracene molecules can produce both homo- and mixdimers upon irradiation. The kinetics of the formation of the mixdimers cannot be determined from the kinetics of the formation of the homodimers directly. Therefore, the comparison of the kinetics of competing photoreactions conducted in the mixture of substrates reveals the relative reactivity of all reacting molecule pairs. It is worth mentioning that the kinetics of photodimerization is very sensitive to the experimental conditions, in particular, the illumination. The importance of the initial conditions is highlighted in the study of Kislyak and co-workers, where even the effect of continuous-wave light sources versus pulsed lasers is discussed [19]. Therefore, simultaneous measurement of kinetic parameters of photodimerization in a mixture is an advantage, as it provides the same experimental conditions for all the reactions and allows us to analyze the relative reactivity in a straightforward manner. The comparison of the photoreactivity of different photoactive compounds is an important issue, and the methodology that can help to reproduce the same illumination conditions is very often addressed in the literature in the context of photoreactions [42].

To conclude the introduction, in the present work, we focus on the investigation of photoreactions in the mixture of anthracenes. To do this, we employ ^1^H NMR spectroscopy, which is being more and more often employed in the field of photochemistry and reaction monitoring [43,44,45,46,47]. We demonstrate that a simple NMR approach can easily deliver data which enables us to obtain insights into the kinetics of the photoreactivity between varying anthracenes. However, as we demonstrate that the NMR approach is easy to employ for photoreaction monitoring, it also has some pitfalls, which will be discussed in this manuscript.

## 2. Results and Discussion

### 2.1. Comparison of A and B in Separate Samples

We begin our research with the investigation of the individual models of **A** and **B.** The choice of **A** is obvious—it can be treated as a reference system, as a large amount of data about the photodimerization of anthracenes is available [2,6,9]. Regarding **B**, it can be easily functionalized via metalation or cross-coupling reactions [48]. Thus, **B** is a good starting point for the development of more functional photoreactive monomers [49]. Moreover, **B** is also utilized as an initiator in the atom transfer radical polymerization (ATRP), which can help to control the polymerization of styrene [23].

**Figure 1:**
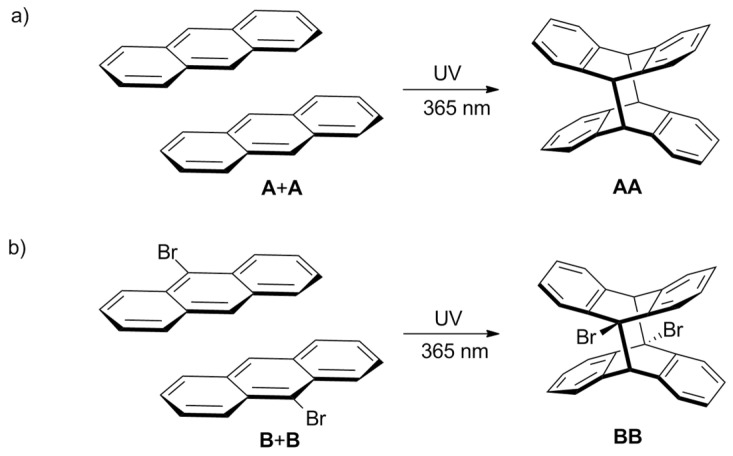
Photodimerization of (**a**) anthracene (**A**) and of (**b**) 9-bromoanthracene (**B**). For **B** only trans isomer is presented as we suppose that this isomer was obtained in the case of our research.

The ^1^H-NMR monitoring of our samples revealed that all investigated compounds form dimers upon 365 nm irradiation, as is typically reported for anthracene and its derivatives (Figure 1). Because of the large aromatic π-surface area, **A** has a relatively low electronic energy gap (π-π*) [8,18]. Therefore, the molecule can easily be excited by irradiation using ultraviolet light. A wavelength of 365 nm was chosen in order to conduct dimerization only, and to avoid the undesirable side reactions, as well as the reverse reaction—the cleavage of a formed dimer [50].

The signals from these homodimers are clearly visible in the ^1^H NMR spectra, which is presented in the next part of the manuscript (see Section 2.3). In the case of **A**, only one dimer is possible, and its signals appear as multiplets at 6.93 and 6.81 ppm, and as the singlet at 4.55 ppm. In the case of **B**, two possible isomers of **B** dimers could be formed. However, the ^1^H NMR spectra have revealed that only one product is present upon illumination in our case. According to the literature data, the dimer with trans located Br atoms (head-to-tail configuration) has been identified in previous studies [22,51]. Therefore, we assume that the resonances of a doublet at 7.76 ppm, a multiplet at 6.96 ppm and a singlet at 5.35 ppm belong to the head-to-tail isomer. It is worth noting that the line at 5.35 ppm is very close to the residual proton signal of the solvent CD_2_Cl_2_ (5.32 ppm) and was not used for qualitative analysis.

**Figure 2:**
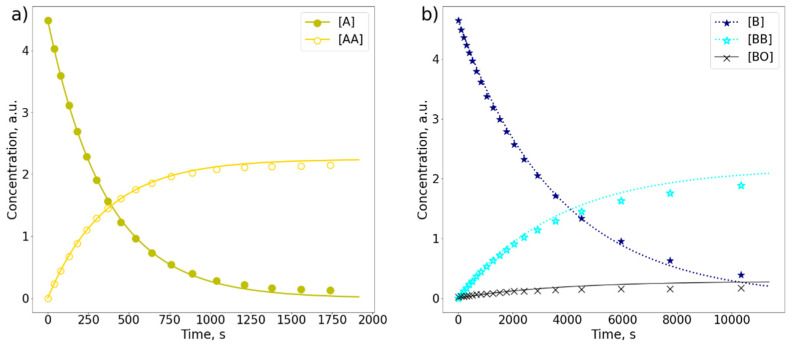
The comparison of photodimerization curves (*λ* = 365 nm) derived from 400 MHz ^1^H-NMR spectra of (**a**) anthracene (**A**) and (**b**) 9-bromoanthracene (**B**) in separate samples each of 4.5 mM concentration in CD_2_Cl_2_. Two products are observed: a dimer of **A** (**AA**) and a dimer of **B** (**BB**).

In order to obtain insights into the photokinetics of dimer formation, changes in the concentration of the substrate and the products during irradiation were analyzed. The NMR monitoring experiments were conducted at substrate concentrations equal to 4.5 mM. The reaction curves derived from ^1^H-NMR spectra are compared in Figure 2. Data were fitted using the following equations:

(1) $$\frac{d\left[A\right]}{dt}=-k_{A}\left[A\right],\;$$

(2) $$\frac{d\left[\mathrm{AA}\right]}{dt}=k_{A}\frac{\left[A\right]}{2}$$

where *k*_A_ is a kinetic constant for a dimerization reaction with **A** and **AA** corresponding to the monomer and the dimer, respectively. In the case of **B**, an additional oxygenation reaction of the monomer occurred, which had to be considered for the kinetic fit. Because of this, the equations had to be modified in this way:

(3) $$\frac{d\left[B\right]}{dt}=-k_{B}\left[B\right]-k_{\mathrm{BO}}\left[B\right],\;$$

(4) $$\frac{d\left[\mathrm{BB}\right]}{dt}=k_{B}\frac{\left[B\right]}{2},$$

(5) $$\frac{d\left[\mathrm{BO}\right]}{dt}=k_{\mathrm{BO}}\left[B\right]$$

where **B** and **BB** correspond to the monomer and the dimer, respectively, while **BO** is the oxidized form of **B**.

The assessed reaction rates revealed that the photodimerization of **B** was about 10 times slower compared to the dimerization process of **A**, with corresponding kinetic constants equal to 2.8 × 10^−3^ s^−1^ and 2.6 × 10^−4^ s^−1^ for **A** and **B,** respectively. It has been noticed that **A** can be almost fully consumed in about 1800 s, while **B** in more than 10,000 s. It has to be stressed that the model we used has been simplified. The reaction is considered to be irreversible, which seems to be a good assumption, because the reverse reaction usually occurs at much higher temperatures or under irradiation with short-wave UV light [3,52,53]. The obtained kinetic data are valid only for our specific irradiation conditions. Therefore, they can be used only as relative numbers showing the difference in reactivity between reacting molecules.

According to the literature, the overall (apparent) reaction order with respect to the anthracene concentration can vary between 0 and 2, depending on the initial conditions [19]. However, S. Dong and co-workers in their review stated that dimerization is a second-order reaction [6]. Moreover, it was also noted that since polymerization is a first-order reaction, competition between polymerization and dimerization should be concentration dependent, and that at low acene concentrations, polymerization is preferred [6]. Based on our results from fitting procedure and analysis of the residual as a function of the reaction order, the minimum of residual for both reactions was at order 1. (Figure S1). Therefore, in our case, the dimerization of both **A** and **B** can be considered as a first-order reaction.

A fitting model included only the dimerization process, assuming that our experimental conditions were set in such a way that all side reactions, as well as oxygenation, could be ignored. The detailed mechanism of dimerization is thoroughly described by Kislyak’s group, including all possible reaction pathways characterized by corresponding individual *k* values [19]. The same group also investigated the equilibrium between dimerization and cleavage of the dimer [50]. They developed a kinetic model, which could be applied under a wide range of reaction conditions including variations in the initial concentrations of **A** and the dimer of **A** (**AA**) as well as λ [50]. According to this group, one of the strengths of a kinetic model if fed with sufficient variations in experimental data is to correct for inconsistencies, as sometimes it is difficult to assess purely experimentally.

The reactivity of anthracenes and their derivatives is influenced by the presence of an electron-withdrawing or electron-donating group in the anthracene unit, as the reactivity of these compounds is mainly determined by their electronic properties [3,52]. **A** can be considered as a reference molecule, where the H atom is present as neither an electron-donating, nor a withdrawing substituent, whereas **B** has an electron-withdrawing Br atom. It is known that the electron-withdrawing groups, also known as deactivating groups, reduce dimerization reactivity in the case of 4 + 2 Diels-Alder cycloaddition [3,53,54]. One can thus speculate that the electron-withdrawing property of the Br atom is the reason why the photodimerization process of B is slower in comparison to the photodimerization of A.

In this study, a few experimental challenges were also encountered, including homogeneous illumination, solubility, oxygenation, and volatility of the solvent and that is the reason why external irradiation of the sample was chosen. There is a variety of in-situ illumination methods presented in the literature [55,56,57] and their advantages compared to ex-situ illumination, especially in terms of experiment time and amount of collected data points, lead to a more accurate evaluation of the reaction mechanism [55]. In spite of this fact, experimental inconsistencies are easier to handle using external irradiation.

### 2.2. The Effect of Experimental Conditions

The easiest parameter to control is the volume of the sample. In order to obtain homogeneous irradiation, it is important to illuminate the entire sample and to avoid convection, which can be expected to change the reaction rate. More details are presented in the supportive information and in Figure S2. The biggest challenge is to obtain the same conditions between different experiments, in particular, the oxygenation level of the samples.

#### 2.2.1. The Effect of the Concentration of Anthracenes

The solubility of **A** and its dimer in acetonitrile, which was used as a solvent, appeared to be too low. This resulted in precipitation after irradiation, which affected the kinetics of the photodimerization. Taking that into account, deuterated dichloromethane (CD_2_Cl_2_) was used as a solvent for the next experiments. First of all, the solubility of A in CD_2_Cl_2_ was significantly higher. Secondly, the π-π and hydrogen bond interactions between the solvent and aromatic anthracenes is not an essential issue in this solvent.

**Figure 3:**
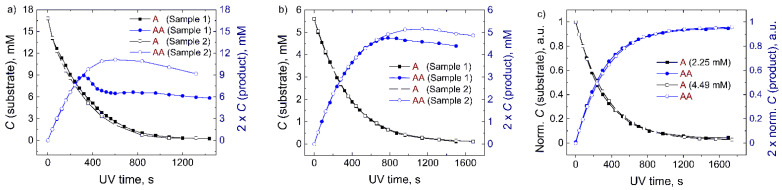
The effect of the precipitation on the photodimerization curves (*λ* = 365 nm) derived from 400 MHz ^1^H-NMR spectra of anthracene (**A**) and its dimer (**AA**). Each experiment was repeated on two separate samples (sample 1 and sample 2) prepared from the same batch. The precipitation was observed (**a**,**b**) until the concentration of anthracene was reduced to 4.5 mM, and no further effect of concentration on kinetics was noticed (**c**).

Based on the visual observations of the solubility of the substrate, the initial concentration of **A** was set to 3 mg/mL (16.8 mM). The reaction curves of two samples (sample 1 and sample 2) derived from the ^1^H-NMR spectra were compared (Figure 3a) in order to check the repeatability of the experiment. At the beginning, the photodimerization processes in both samples occurred in the same manner. However, after the consumption of about 50% of the substrate, the kinetics of the dimer formation started to differ. The results suggested that the solubility of the product was not sufficient. The precipitation of the product could also be detected visually. Hence, the concentration of **A** was further reduced (Figure 3) until no precipitation of the product was observed, neither visually nor from the reaction curves.

The comparison of two separate experiments for the same system resulted in the same decay curve only when the initial concentration of **A** was 4.5 mM (0.8 mg/mL) or lower. Moreover, the further dilution of the sample did not show any significant effect on the kinetics of the photodimerization. The comparison of the kinetics when the concentration of **A** was 4.5 mM and 2.25 mM is demonstrated in Figure 3c. It is worth mentioning that the irradiation curves shown in Figure 3 allow us to estimate the solubility of the reaction product. In this case, the value of about 4.5 mM corresponds to a saturated solution of the anthracene dimer (**AA**).

Crystallization of the product can be directly observed in the NMR sample tube on the walls, which were directly irradiated (Figure S3). This shows that during the illumination, a concentration gradient of products and substrates is present in the solution. The sample volume is not stirred, and therefore, the estimated values of the solubility of the product described above could contain an error.

An analogous trend was observed for **B**. This means that the system works in a fairly repeatable manner if the oxygen level in the samples is maintained the same. Low pressure/vacuum (LPV) NMR tubes equipped with a J. Young valve not only allowed us to solve the issue of the volatile solvent, but also helped us to eliminate or significantly reduce the risk of oxygen getting into the sample.

#### 2.2.2. The Effect of Oxygenation of the Sample

According to the literature, anthracene exposed to air and light would also undergo an oxidation reaction to yield EPOs. The mechanism of these kinds of photochemical oxidation reactions proceeds via two possible routes. The first is based on the electron transfer from the first excited singlet state of **A** to the oxygen in the ground state. The other pathway involves a triplet state of **A** (^3^A) and is based on energy transfer. During this mechanism, a triplet sensitizer is usually required to transfer its energy from ^3^A to ^3^O_2_ to form singlet oxygen ^1^O_2_, which subsequently reacts with acene to form EPOs [6]. Most research is done on the shorter acenes because longer conjugations lead to faster oxidation by air, and thus, the purification of the post-reaction mixture is more difficult [18]. Several groups have utilized density functional theory (DFT) calculations, showing that the reactivity of acenes originates from the increased diradical character of longer acenes [6].

**Figure 4:**
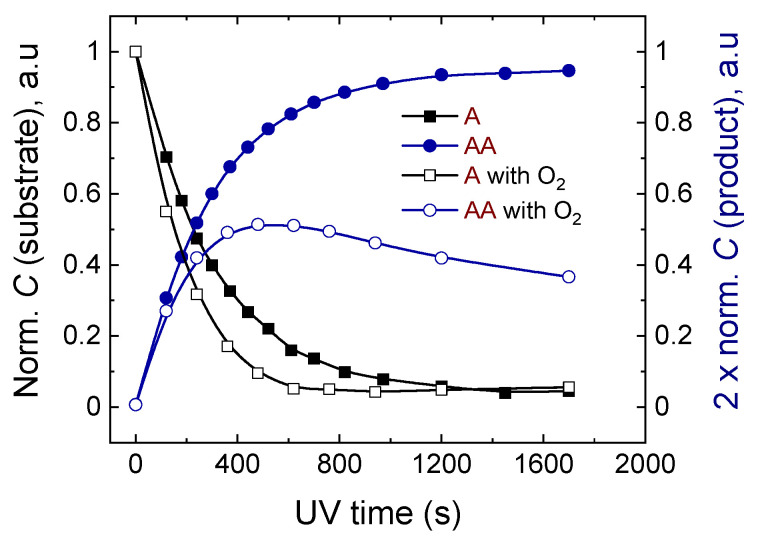
The effect of oxygenation of the sample on the photodimerization curves (*λ* = 365 nm) derived from 400 MHz ^1^H-NMR spectra of anthracene (**A**).

**Figure 5:**
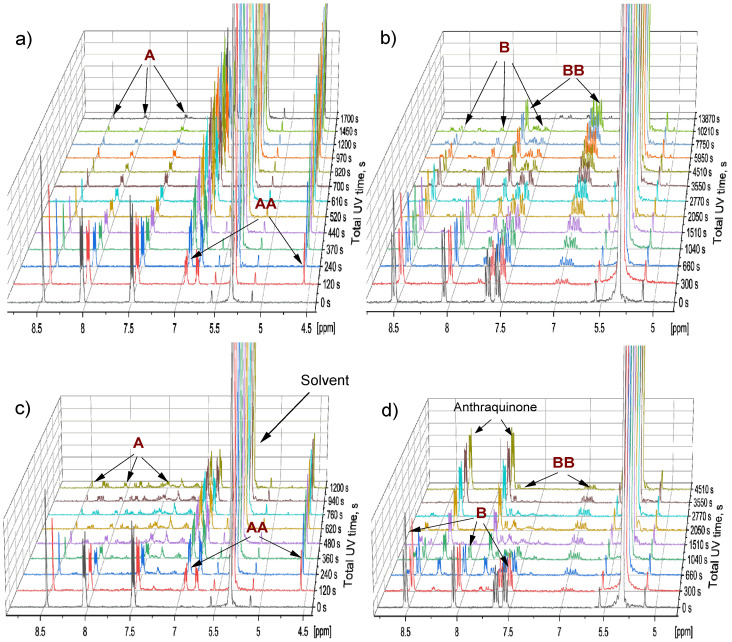
The 400 MHz ^1^H-NMR spectra of anthracene (**A**), dimer of anthracene (**AA**), 9-bromoanthracene (**B**) and dimer of 9-bromoanthracene (**BB**) in: (**a**,**b**) deoxygenated samples and (**c**,**d**) samples with oxygen at different reaction times, i.e., total time of UV illumination (λ = 365 nm).

To investigate the effect of oxygen, two samples of **A** and **B** (2.25 mM) were deliberately exposed to oxygen by opening an NMR tube for about 30 s. The results were compared to those derived from the identical deoxygenated samples. A completely different behavior was observed for oxygenated and oxygen-free samples. The presence of oxygen could be tracked by comparing the consumption of the substrate in oxygenated and deoxygenated samples, which can be seen from the reaction curves (Figure 4), as well as directly from the ^1^H-NMR spectra (Figure 5). In the cases of both **A** and **B**, it is seen that the substrate is consumed significantly faster in the samples with oxygen compared to the ones without exposure to oxygen.

Moreover, as witnessed by the reaction curves of **A** and its dimer **AA**, only about 50% of the substrate converts to the product, and at the end of the photodimerization reaction, a slight reappearance of the substrate as well as consumption of the dimer is observed. The effect of oxygenation could also be observed by the appearance of additional peaks in the ^1^H-NMR spectra (Figure 5). Two resonances from anthraquinone were observed at 7.83 and 8.29 ppm. The peaks were confirmed by registering the ^1^H-NMR spectra of anthraquinone (Figure S4). For more details, please see the Supporting Information (SI, pages 4–6). The existence of anthraquinone suggests that EPOs were generated in our sample, as EPO decomposition leads to the anthraquinone systems [15].

Supported by the results, it can be concluded that evaluation of the required experimental conditions and their influence on the photochemical reactions of anthracenes is essential in order to ensure that the entire setup works in a repeatable manner. This means that the quantitative data can be compared for systems with different compounds.

### 2.3. Comparison of A and B in the Mixture

The reaction rate calculated for the sample containing only one compound will be different under different experimental setups. This is because the maintenance of the same experimental conditions is sometimes difficult to achieve. Thus, in order to develop a more reliable approach, for the comparison of the photoreactivity of various anthracenes, we further moved on to experiments with mixtures what is the main part of this study.

**Figure 6:**
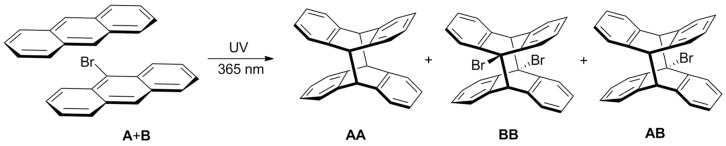
Photodimerization of anthracene and 9-bromoanthracene in the mixture. The reaction gives three possible products as a dimer **AA**, **AB** and **BB**.

Samples containing two anthracene derivatives were investigated. Such an approach seemed to be a more reliable one, as the dimerization of both substrates took place in the same sample, meaning that photoreactions were monitored under the exact same conditions. Moreover, in the system with two compounds, the formation of three products was observed in the NMR spectra: the dimer of **A** (**AA**), the dimer of **B** (**BB**) and the dimer of **A** and **B** (**AB**) (Figure 6).

**Figure 7:**
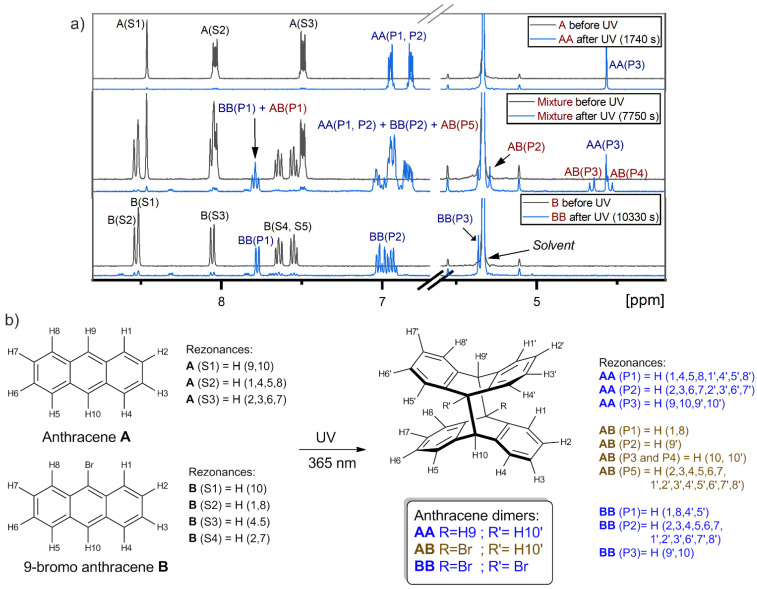
(**a**) The comparison of 400 MHz ^1^H-NMR spectra of 2.25 mM of pure anthracene (**A**) (at the top), 2.25 mM of pure 9-bromoanthracene (**B**) (at the bottom) and their mixture (in the middle) before (upper black spectrum) and after (lower blue spectrum) UV illumination (*λ* = 365 nm) as well as (**b**) the assignment of the spectral lines. S1–S5 and P1–P5 represent spectral lines of substrate and product respectively. **AA**, **BB** and **AB** correspond to the dimer of anthracene, dimer of 9-bromoanthracene and dimer of anthracene and 9-bromoanthracene, respectively. Samples were prepared in deuterated CD_2_Cl_2_ in the glove box.

The resonances of **AA** and **BB** appeared at the same ppm as in the samples with one compound, only this time overlapping with each other or with the resonances from **AB**. The doublet of **AB** at 7.78 ppm (**AB**(P1)) interferes with the doublet of **BB** at 7.76 ppm (**BB**(P1)). The signal from the aromatic part of AB overlaps with the multiplet of **BB** at 6.96 ppm (**BB**(P2)) as well as with the signals from **AA** at 6.93 ppm (**AA**(P1)) and 6.81 ppm (**AA**(P2)). Similar to the singlet of **BB** at 5.35 ppm (**BB**(P3)), a singlet of **AB** is also observed close to the solvent peak at 5.27 ppm (**AB**(P2)). Finally, the doublet of doublets at 4.64 ppm (**AB**(P3)) and 4.52 ppm (**AB**(P4)) from the protons at the 10 and 10′ positions of the dimer **AB** (Figure 7) appear at the same region as singlet of **AA** at 4.55 ppm (**AA**(P3)), for which the integral can be calculable, even if the signals are slightly overlapped.

**Figure 8:**
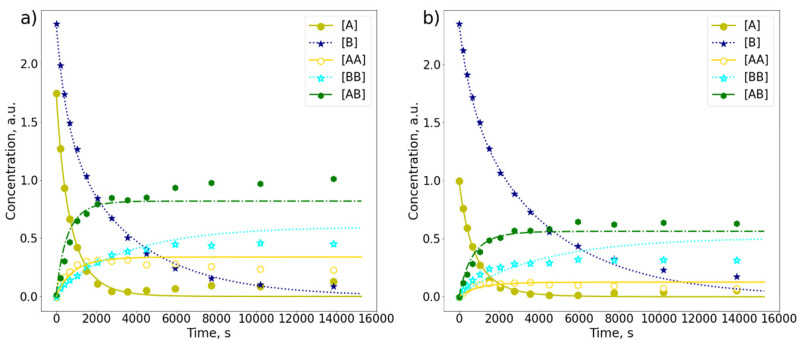
The comparison of photodimerization curves (*λ* = 365 nm) derived from 400 MHz ^1^H-NMR spectra of anthracene (**A**) and 9-bromoanthracene (**B**) in the mixture with molar mixing ratios (**a**) 1:1.3 and (**b**) 1:2.3. Three dimmers are observed: **AA**, **BB** and **AB**.

The analysis of mixture kinetics was more complex because most of the chemical shifts of **AB** protons were either in the same range as chemical shifts of **AA** and **BB** dimers, or too close to the solvent peak to be distinguishable. Two types of mixture with a molar ratio of **A** to **B** equal to 1:1.3 (Figure 8a) and 1:2.3 (Figure 8b) were investigated.

When analyzing the data, we realized that apart from the creation of homodimers and mixed dimers there were some other reactions occurring that could not be tracked by NMR in a sense, that their resonances are low, and the integration is burdened with large errors. However, if we expect that the mass of the system (mass of substrates and products) is constant, then from the loss of the mass of the system, we can track the side product creations. To compensate for these side-reactions, we have assumed that all mass loss of **A**, including the oxygenation effect, is responsible for the creation of unknown compound **XA** and similarly for **B** the **XB** compound is formed. The **XA** and **XB** concentration was calculated using such equations:

(6) $$\left[\mathrm{XA}\right]={\left[A\right]}_{0}+{\left[\mathrm{AA}\right]}_{0}+{\left[\mathrm{AB}\right]}_{0}-\left[A\right]-\left[\mathrm{AA}\right]-\left[\mathrm{AB}\right],$$

(7) $$\left[\mathrm{XB}\right]={\left[B\right]}_{0}+{\left[\mathrm{BB}\right]}_{0}+{\left[\mathrm{AB}\right]}_{0}-\left[B\right]-\left[\mathrm{BB}\right]-\left[\mathrm{AB}\right]$$

where A_0_, AA_0_, AB_0_ and B_0_ stand for the initial concentrations.

It was also noticed that in some cases, for longer irradiation times, reactions are slightly reversible, which might be caused by the solvent, or by the changes in the temperature due to longer illumination times of up to few hours or by some other yet-unexplored effects. However, since it does not have a significant impact on the comparison of the kinetics of our compounds, it was decided to leave the investigation for further studies. Hence, for the simplicity of the model, such reversible reactions were not included in the calculations and the data presented in Figure 8a,b was fitted using the first 6000 s with the system of differential equations:

(8) $$\frac{d\left[A\right]}{dt}=-k_{A}\left[A\right]-k_{\mathrm{AB}}{\left[A\right]}^{\frac{1}{2}}{\left[B\right]}^{\frac{1}{2}}-k_{\mathrm{XA}}\left[A\right],$$

(9) $$\frac{d\left[B\right]}{dt}=-k_{B}\left[B\right]-k_{\mathrm{AB}}{\left[A\right]}^{\frac{1}{2}}{\left[B\right]}^{\frac{1}{2}}-k_{\mathrm{XB}}\left[B\right],$$

(10) $$\frac{d\left[\mathrm{AA}\right]}{dt}=k_{A}\frac{\left[A\right]}{2},$$

(11) $$\frac{d\left[\mathrm{BB}\right]}{dt}=k_{B}\frac{\left[B\right]}{2},$$

(12) $$\frac{d\left[\mathrm{AB}\right]}{dt}=k_{\mathrm{AB}}{\left[A\right]}^{\frac{1}{2}}{\left[B\right]}^{\frac{1}{2}},$$

(13) $$\frac{d\left[\mathrm{XA}\right]}{dt}=k_{\mathrm{XA}}\left[A\right],$$

(14) $$\frac{d\left[\mathrm{XB}\right]}{dt}=k_{\mathrm{XB}}\left[B\right].$$

Here *k*_A_, *k*_B_, *k*_AB_, *k*_XA_ and *k*_XB_ are the kinetic constants for the reactions **A** + **A** → **AA**, **B** + **B** → **BB**, **A** + **B** → **AB**, **A** → **XA** and **B** → **XB**, respectively. The calculated *k*_A_, *k*_B_ and *k*_AB_ values are gathered in Table 1. In the case of the mixture, *k*_A_ and *k*_B_ values were higher when mixing molar ratio corresponded to the more comparable concentrations of **A** and **B**. For instance, when the mixing ratio was 1:1.3 for **A**:**B**, *k*_A_ and *k*_B_ were equal to 5.01 × 10^−4^ s^−1^ and 2.15 × 10^−4^ s^−1^, respectively, while accordingly at mixing ratio of 1:2.3 the *k*_A_ and *k*_B_ values dropped to 3.23 × 10^−4^ s^−1^ and 1.43 × 10^−4^ s^−1^, respectively. However, the ratio *k*_A_/*k*_B_ remained the same and was equal to approximately 2. The same ratio estimated from the measurements carried out in the solutions with only **A** or **B** was about 5 times larger. Nevertheless, the same trend was observed, confirming that **A** reacts faster than **B**. Moreover, as we can see, the kinetic constant *k*_AB_ corresponding to the formation of the mixdimer **AB** remained the same for both studied mixtures.

Finally, regarding the more comprehensive discussion of the obtained data, an important aspect has to be mentioned. When two different anthracene molecules **A** and **B** are involved in a photoreaction, two scenarios are possible: A* + **B** → **AB** or **A** + B*→ **AB**, where A* and B* stand for molecules in the excited electronic states. NMR spectra do not give enough information to determine which scenario takes place or dominates. In this context, the use of optical spectroscopy and quantum chemistry calculations could significantly support NMR. To conclude, the answer to this question requires more comprehensive studies which are beyond the scope of the present article.

## 3. Materials and Methods

### 3.1. Samples

The solution of anthracene (**A**) and 9-bromoanthracene (**B**) were chosen for monitoring the photodimerization process. Both substrates were bought from Sigma Aldrich and dissolved in CD_2_Cl_2_ (99.6% D), which was purchased from Deutero GmbH. An additional compound, anthraquinone, which was used for the confirmation of spectral lines when the oxygenation process was analyzed, was bought from TCI Chemicals. All chemicals were used as received from the supplier without any further purification. The NMR samples were prepared in a glove box under inert gas conditions. The samples of 0.48 mL volume were placed in 5 mm low pressure/vacuum (LPV) NMR tubes equipped with J. Young valves to avoid oxygenation, changes of the moisture content and evaporation of the solvent. Unless stated otherwise, the concentration of our substrates was either 4.5 mM or 2.25 mM.

### 3.2. UV Illumination

UV illumination was performed by a 365 nm (9 nm FWHM) collimated LED (M365LP1-C4) with a beam diameter of 44 mm purchased from Thorlabs. It contained a 2.5 mm × 2.5 mm mounted LED with a maximum power of 2000 mW, and an irradiance of 21.0 µW/mm^2^ when operating at a 1700 mA maximum current. An approximate total beam power of collimated LED; however, it was reduced to 615 mW (1700 mA) after passing through the aspheric collimation optic. During all experiments, the LED current was set to 700 mA using a T-cube LED driver (LEDD1B), also bought from Thorlabs. An NMR tube containing 480 μL of the solution was placed 14 cm away from the LED’s lens, resulting in the full illumination of the sample before each NMR measurement. The duration of UV irradiation varied from 60 s to 60 min depending on the stage and the reaction rate of the photodimerization process.

### 3.3. NMR Experiment

NMR experiments were carried out at room temperature on a VARIAN VNMRS Oxford AS400 NMR spectrometer operating at the resonance frequency of 400 MHz for ^1^H (magnetic field of 9.4 T) using an ASW dual-resonance broadband gradient NMR probe head. The ^1^H-NMR monitoring of each individual compound was performed at least twice as separate experiments to ensure that the experimental setup works in a repeatable manner. The signal of tetramethylsilane was used as a reference. The 90° pulse length was 10.75 μs, and 8 scans were accumulated with a repetition delay of 40 s. The spectral width was 12 ppm with 16k points as the size of the FID. Relaxation times, *T*_1_, were measured by the inversion recovery method. The NMR spectra were processed using TopSpin 4.0.7 software.

### 3.4. Data Analysis

The concentration of substrates and products in the investigated samples was deduced from the integrals of the best distinguishable ^1^H-NMR spectral lines measured after each UV illumination. Then, the reaction curves were derived showing the concentration dependence on total UV irradiation time, which corresponds to the reaction time since, the reaction occurs only during the illumination. The reaction law for one compound can be described by the following equation:

(15) $$\frac{d\left[C\right]}{dt}=k{\left[C\right]}^{x}$$

where *k* is the rate constant, [C] is the concentration of substrate C in the photodimerization reaction and *x* refers to the reaction order with respect to the reactant C. All the fittings were performed using the Symfit library in Python [58]. The order of the reactions was established using data from single compound experiments. The fits of the reaction order from 0 to 3 were performed with a step of 0.1 followed by the analysis of the residual as a function of reaction order. The minimum value of the residual corresponded to the most probable reaction order with respect to the selected compound, as illumination conditions and sample.

## 4. Conclusions

Despite the recent progress in the photochemistry of anthracenes, the photosynthesis of novel materials is still a demanding task which necessitates a deeper understanding of the photochemistry of this class of aromatic compounds. Here, we demonstrate that the photoreactivity of different anthracenes can be investigated via NMR in a mixture. To the best of our knowledge, the kinetics of such mixtures has not been investigated so far. In the case of a mixture of anthracene (**A**) and 9-bromoanthracene (**B**), two homodimers **AA** and **BB**, and one mixdimer **AB** were identified by ^1^H NMR upon the UV irradiation. The ^1^H NMR enables the monitoring of the photoreaction progress. This approach allows one to study the kinetics of three photoreactions simultaneously in the same solution, i.e., under exactly the same conditions. Thus, the relative reactivity obtained in this way is more informative. Specifically, the kinetics data show that the **AB** formation can be as fast as the **AA** formation, which is not obvious, as **B** contains bromide as a deactivating group. This shows that the mixdimers formation seems to be somehow favored over homodimers. This is important because as the mixdimer is predominant in comparison to the two homodimers, the photofabrication of materials based on the mixdimers can be greatly facilitated.

Finally, the presented approach can be treated as a methodology for the more comprehensive investigation of the photoreactivity of varying anthracenes, as we have evaluated the required experimental conditions and their influence on the photochemical reactions of anthracenes. In particular, oxygen can blur the picture of the kinetics, however, the adverse effect of oxygen can be included in the kinetic model, which improves the reliability of the kinetic data. The obtained knowledge opens up an easy and reliable route for further development of the in-situ illuminated-NMR monitoring system, which requires a more sophisticated illumination setup in terms of identical experimental conditions. Moreover, the integration of the in-situ illuminated-NMR with other spectroscopic methods, for example, fluorescence, can be a powerful tool that supports the NMR investigation of the photodimerization of varying anthracenes.

## Figures and Tables

**Table 1:** Kinetic constants of photodimerization of anthracene (**A**) and 9-bromoanthracene (**B**) in separate samples and in the mixtures. Values *k*_A_, *k*_B_ and *k*_AB_ were calculated using a fitting procedure for the first 6000 s and correspond to reactions **A** + **A** → **AA**, **B** + **B** → **BB**, **A** + **B** → **AB**, respectively.

Sample	*k*_A_, s^−1^	*k*_B_, s^−1^	*k*_AB_, s^−1^
**A** (4.5 mM)	(2.8 ± 0.03) × 10^−3^	-	-
**B** (4.5 mM)	-	(2.60 ± 0.06) × 10^−4^	-
Mixture of **A**:**B** = 1:1.3	(5.01 ± 0.83) × 10^−4^	(2.15 ± 0.26) × 10^−4^	(3.88 ± 0.41) × 10^−4^
Mixture of **A**:**B** = 1:2.3	(2.81 ± 1.04) × 10^−4^	(1.32 ± 0.19) × 10^−4^	(3.89 ± 0.59) × 10^−4^

## Data Availability

The raw dataset is published in zenodo.org and is available at: https://doi.org/10.5281/zenodo.5543208.

## Supplementary Materials

The following are available online, Figure S1: determination of photodimerization reaction order, Figure S2: the effect of illuminated volume of the sample, Figure S3: crystallization of the photodimerization product, Scheme S1: The general scheme of photooxygenation, Figure S4: ^1^H-NMR spectra of anthraquinone, Figure S5: Photodimerization of anthracenes A and B under inert gas atmosphere, Figure S6: the comparison of photodimerization curves of **A** and **B** in separate samples, and in the mixture when fitting was done using the first 6000 s, and the model included the product of oxygenation as well as unidentified products, Figure S7: ^1^H-NMR spectra of **A** and **B** in separate samples, and in the mixture using benzene as a solvent, Table S1: kinetic constants *k*_XA_, *k*_XB_ and *k*_BO_.
