# Supplementary material for: Photoreactivity of an Exemplary Anthracene Mixture Revealed by NMR Studies, including a Kinetic Approach
Source: Molecules. 2021 Nov 5;26(21):6695. doi: 10.3390/molecules26216695 (PMC8587725; doi:10.3390/molecules26216695)
Supplement: Supplementary file 1 [file molecules-26-06695-s001.zip › molecules-1426143-supplementary.pdf]

Photoreactivity of an Exemplary Anthracene Mixture Revealed by NMR Studies, Including a Kinetic Approach

Kristina Kristinaityte, Mateusz Urbanczyk, Adam Mames, Mariusz Pietrzak* and Tomasz Ratajczyk*

Polish Acad Sci, Inst Phys Chem, PL-01224 Warsaw, Poland;
* Correspondence: mpietrzak@ichf.edu.pl; tratajczyk@ichf.edu.pl;

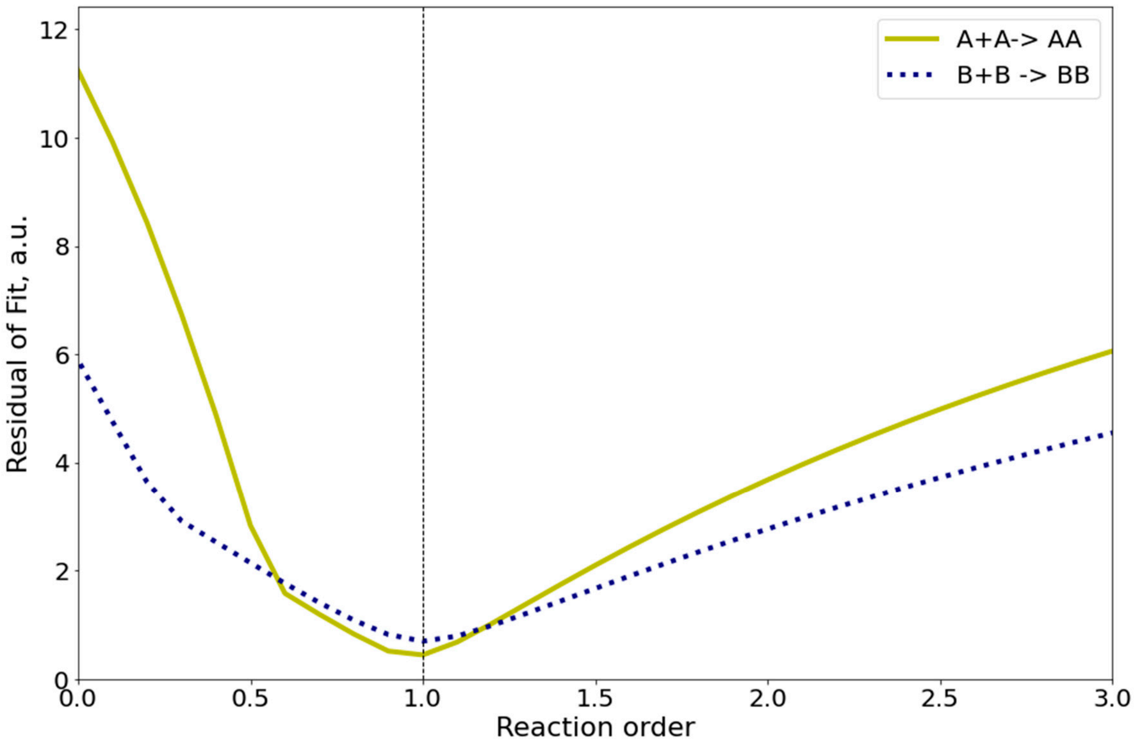

Figure S1. Determination of photodimerization reaction order in respect with anthracene (**A**) and 9-bromoanthracene (**B**).

The effect of illuminated sample volume

Volume of illuminated sample is the main factor when homogeneous irradiation is the goal employing outside illumination. Depending on the LED beam diameter and illumination set-up, the amount of the sample in our case could not exceed 480 µl, in order to be fully illuminated. When the volume of the sample is larger it is illuminated only partially. Therefore, the convection takes place and it is expected that the reaction rate will significantly decrease.

This was confirmed by illuminating different amount of otherwise identical samples under the same experimental conditions. The consumption of **A** as well as the formation of **AA** dimer was 2 times slower when only about half of the sample was illuminated (900 µl) compared to full illumination (480 µl) (Figure S2). For illustrative purpose, the concentration of the product was doubled so that it would be easier to evaluate the conversion between the substrate and the dimer. The results indicate that even a slight change in illuminated volume due to a different amount of the sample or different illumination conditions will lead to different reactivity of the substrate.

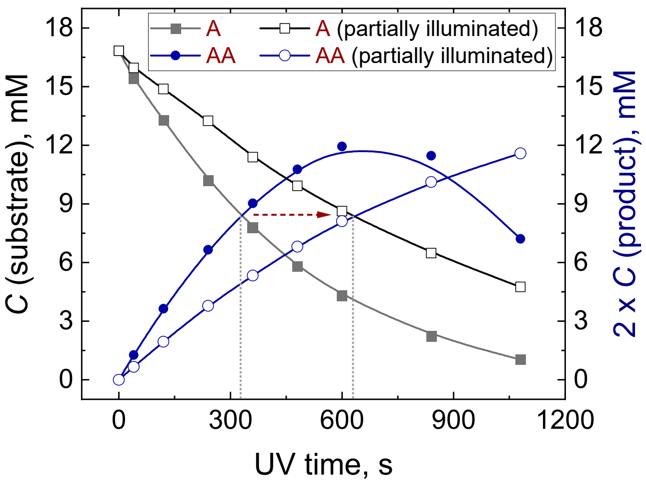

Figure S2. The effect of illuminated volume of the sample on the photodimerization curves (λ = 365 nm) derived from 400 MHz ^{1}H-NMR spectra of anthracene (**A**).

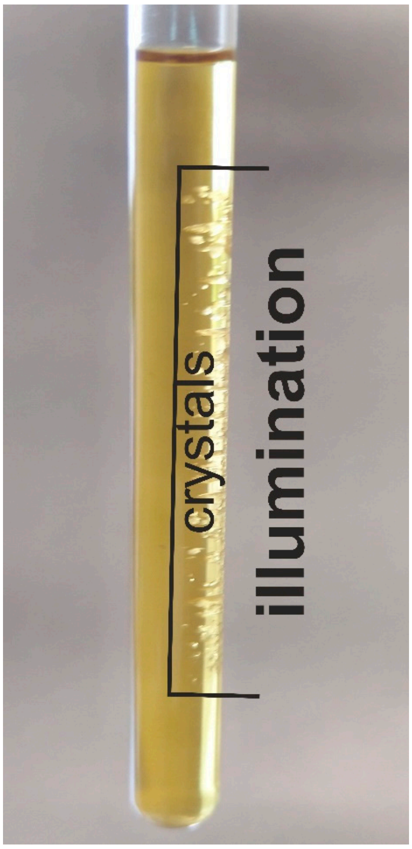

Figure S3. Crystallization of the photodimerization product observed in the NMR sample tube of 9-bromoanthracene solution after the direct UV illumination (λ = 365 nm).

The influence of oxygen on the photodimerization.

It is well known that upon irradiation, anthracenes can react with oxygen [1]. This reaction yields mainly anthracene endoperoxides. However, other products of reactions are also possible. While the mechanism of reactivity between anthracenes and oxygen is well known, the reactivity of endoperoxides -in particular, their decomposition is still not fully understood. However, it has already been established that decomposition leads to anthraquinone [2]. Interestingly, the decomposition of 9-substituted and 9,10-disubstituted anthracene EPOs also yields anthraquinone. In our sample, anthraquinone was identified, which suggests that anthracene endoperoxide (EPO) was present in our sample. (Scheme S1).

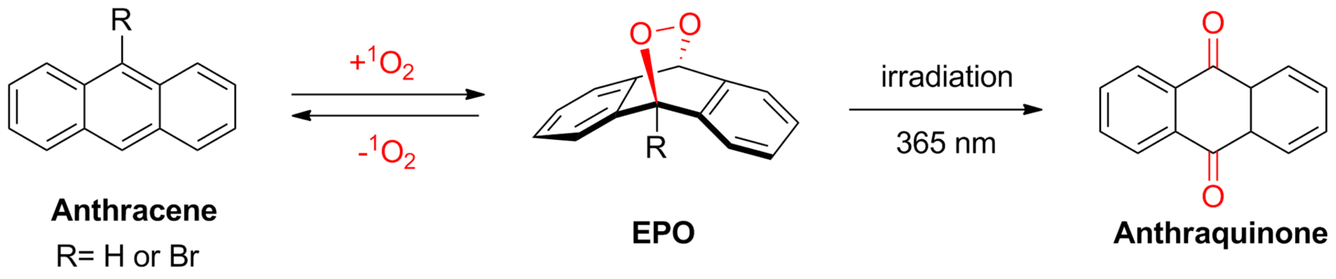

Scheme S1. The general scheme of photooxygenation between anthrecenes derivatives and singlet oxygen. The formation of Anthraquinone via Endoperoxides (EPOs) as an intermediate.

Comparing our two experiments: photodimerization with oxygen (Figure S4) and photodimerization under inert gas conditions (Figure S5), it can be said that the oxidation of anthracenes is an undesirable competitive reaction. No anthraquinone formation from anthracene **A** was observed under inert conditions. In the case of photodimerization of 9-bromoanthracene **B**, the formation of anthraquinone was visible, but its amount was negligible and this did not prevent us interpreting the results.

[1] P. P. Fu, Q. Xia, X. Sun, and H. Yu, "Phototoxicity and Environmental Transformation of Polycyclic Aromatic Hydrocarbons (PAHs)—Light-Induced Reactive Oxygen Species, Lipid Peroxidation, and DNA Damage," *J. Environ. Sci. Heal. Part C*, vol. 30, no. 1, pp. 1–41, Jan. 2012, doi: 10.1080/10590501.2012.653887.

[2] M. Klaper, P. Wessig, and T. Linker, "Base catalysed decomposition of anthracene endoperoxide," *Chem. Commun.*, vol. 52, no. 6, pp. 1210–1213, 2016, doi: 10.1039/C5CC08606J.

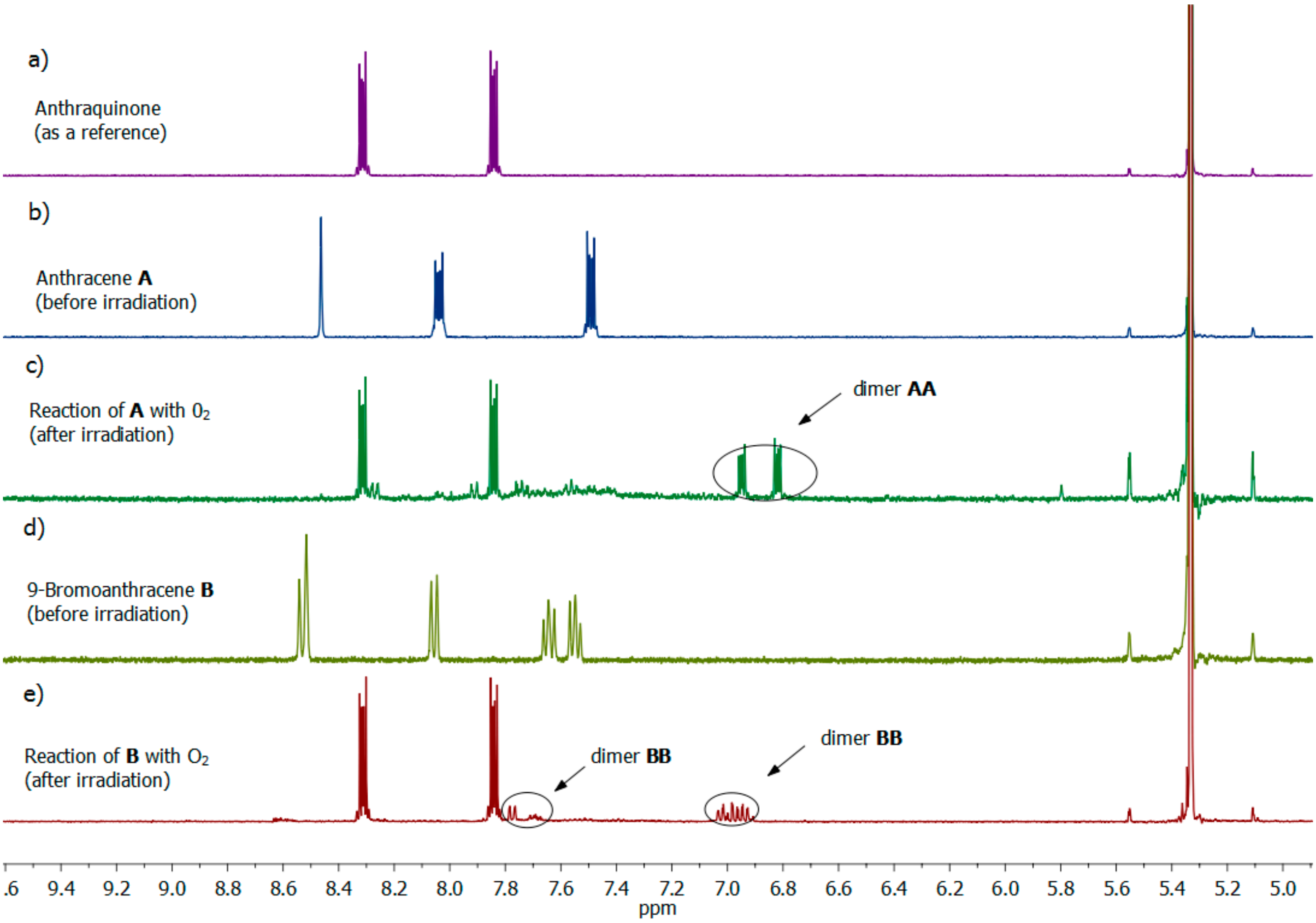

Figure S4. Reaction of anthracenes **A** and **B** with oxygen under irradiation conditions. Samples A and B were prepared in exactly the same way. a) ^{1}H NMR (400 MHz, CD_2Cl_2) of anthraquinone as a reference. b) ^{1}H NMR (400 MHz, CD_2Cl_2) of anthracene **A** before irradiation. c) ^{1}H NMR (400 MHz, CD_2Cl_2) of anthracene **A** after 1 hour of irradiation with the presence of oxygen. d) ^{1}H NMR (400 MHz, CD_2Cl_2) of 9-bromoanthracene **B** before irradiation. e) ^{1}H NMR (400 MHz, CD_2Cl_2) of 9-bromoanthracene B after 2 hours of irradiation with the presence of oxygen.

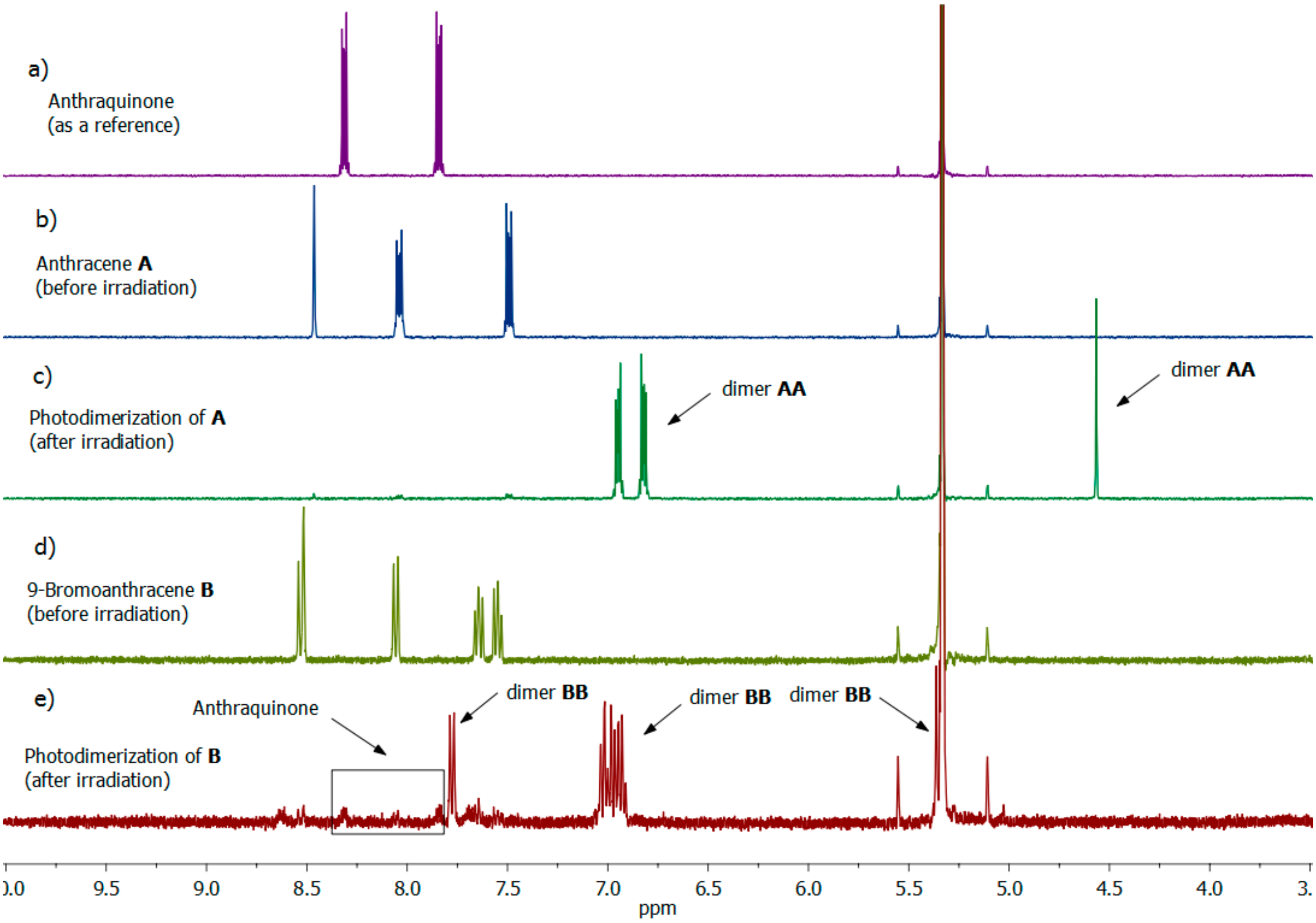

Figure S5. Photodimerization of anthracenes **A** and **B** under inert gas atmosphere. Samples **A** and **B** were prepared in exactly the same way. (in the glovebox) a) ^{1}H NMR (400 MHz, CD_2Cl_2) of anthraquinone as a reference. b) ^{1}H NMR (400 MHz, CD_2Cl_2) of anthracene **A** before irradiation. c) ^{1}H NMR (400 MHz, CD_2Cl_2) of anthracene **A** after 1 hour of irradiation under inert gas atmosphere. d) ^{1}H NMR (400 MHz, CD_2Cl_2) of 9-bromoanthracene **B** before irradiation. e) ^{1}H NMR (400 MHz, CD_2Cl_2) of 9-bromoanthracene **B** after 2 hours of irradiation under inert gas atmosphere.

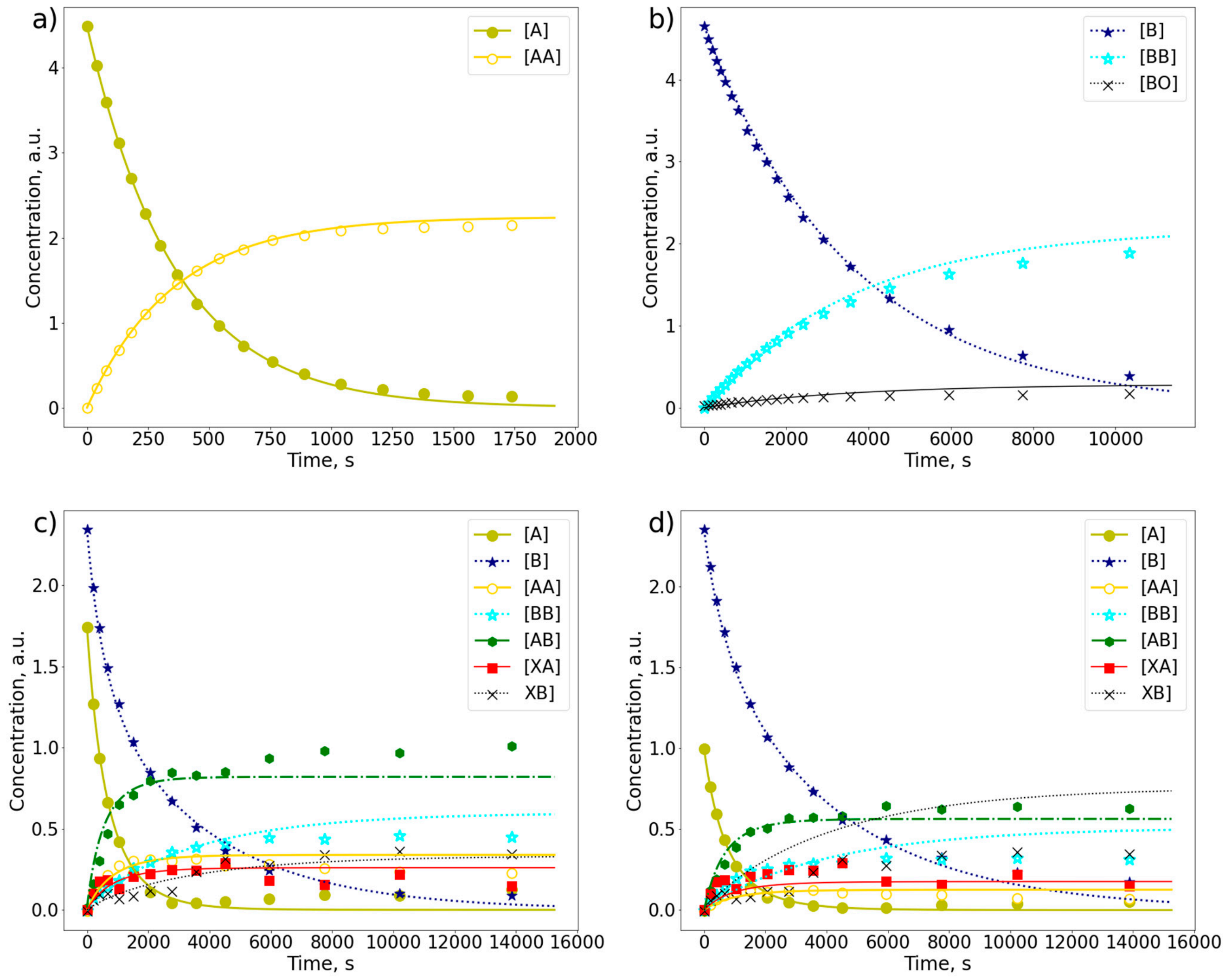

Figure S6. The comparison of photodimerization curves (λ = 365 nm) derived from 400 MHz ^{1}H-NMR spectra of a) anthracene (**A**) and b) 9-bromoanthracene (**B**) in separate samples each of 4.5 mM concentration in CD_2Cl_2 as well as in the mixture with molar mixing rations c) 1:1.3 and d) 1:2.3. Three dimers are observed: **AA**, **BB** and **AB**. **XA** and **XB** are unidentified compounds responsible for all mass loss of the anthracene (**A**) and 9-bromoanthracene (**B**) respectively. **BO** stands for the product of oxygenation. Data was fitted using the first 6000 s.

Table S1. Kinetic constants k_{XA}, k_{XB} and k_{BO} of photodimerization of anthracene (**A**) and 9-bromoanthracene (**B**) in separate samples and in the mixtures, where **XA** and **XB** are unidentified compounds responsible for all mass loss of the anthracene (**A**) and 9-bromoanthracene (**B**) respectively, while **BO** stands for the product of oxygenation. Data was calculated using fitting procedure for the first 6000 s.

**Sample**	**k_{XA}, s^{-1}**	**k_{XB}, s^{-1}**	**k_{BO}, s^{-1}**
B (4.5 mM)	-		(0.17 ± 0.03) x 10^{-4}
Mixture of A:B = 1:1.3	(1.90 ± 0.46) x 10^{-4}	(0.57 ± 0.57) x 10^{-4}	-
Mixture of A:B = 1:2	(1.97 ± 0.7) x 10^{-4}	(0.98 ± 0.12) x 10^{-4}	-

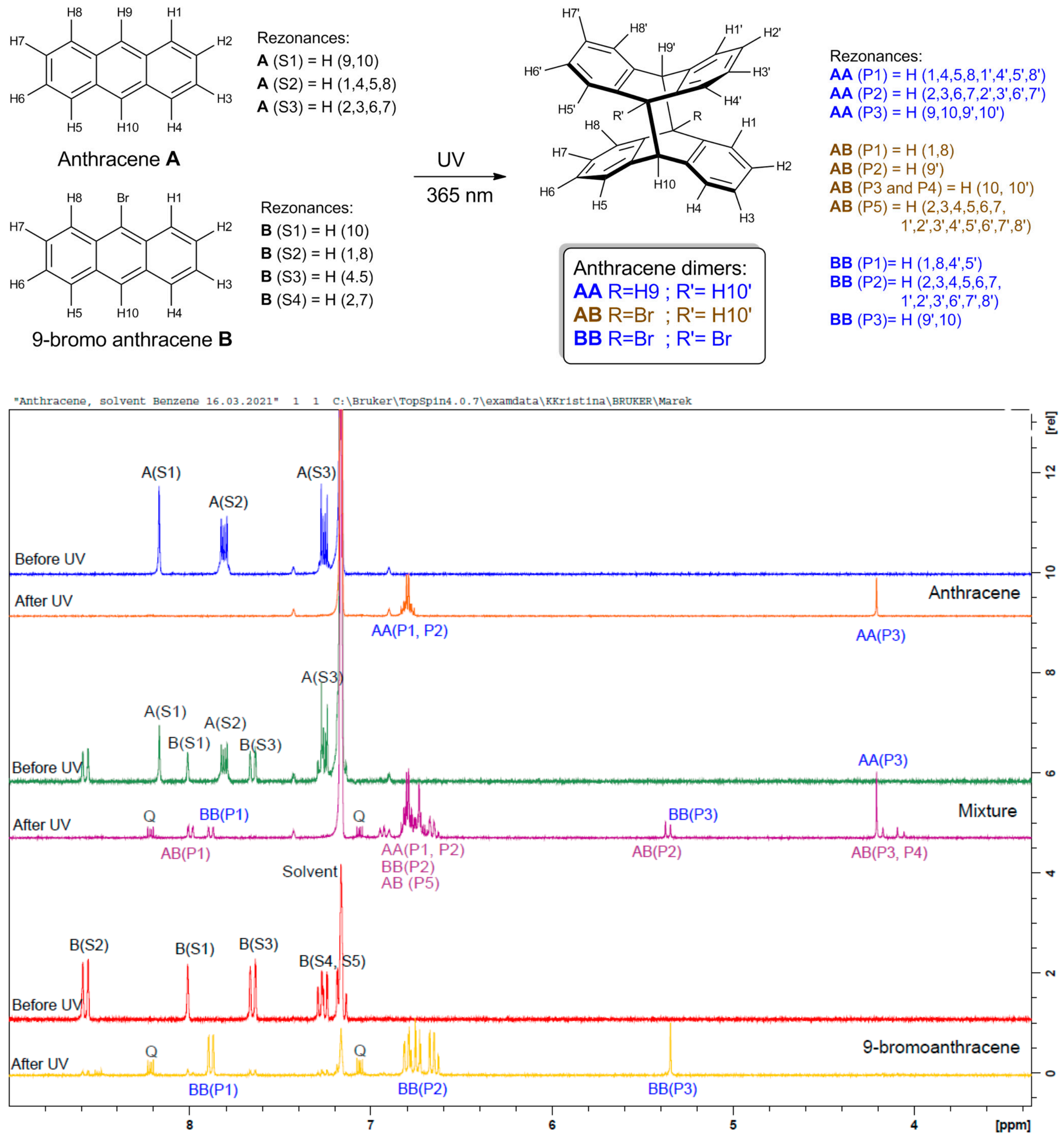

Figure S7. The comparison of zoomed 400 MHz ^{1}H-NMR spectra of 4.5 mM of pure anthracene (**A**) (at the top), 4.5 mM of pure 9-bromoanthracene (**B**) (at the bottom) and their mixture (in the middle) before (upper spectrum) and after (lower spectrum) UV illumination (λ = 365 nm) as well as the assignment of the spectral lines. S1-S5 and P1-P3 represents spectral lines of substrates and products respectively. **AA**, **BB** and **AB** correspond to a dimer of anthracene, a dimer of 9-bromoanthracene and a dimer of anthracene and 9-bromoanthracene respectively, **Q** corresponds to antraquinone, the product of oxigenation. Samples were prepared in Benzene-d$_6$ (99.5% or 99.96%) in the glove box.
